# Scleral Transillumination-Guided Trocar Placement During Vitrectomy in Highly Myopic Eyes: A Case Report

**DOI:** 10.7759/cureus.110575

**Published:** 2026-06-10

**Authors:** Keigo Takagi, Hinako Ohtani, Chisako Ida, Kazunobu Sugihara, Masaki Tanito

**Affiliations:** 1 Department of Ophthalmology, Shimane University Faculty of Medicine, Izumo, JPN

**Keywords:** high myopia, scleral transillumination, surgical procedure, trocar placement, vitrectomy

## Abstract

High myopia can make pars plana vitrectomy technically challenging because axial elongation may increase the distance between the conventional trocar insertion site and the posterior pole. Accordingly, more posterior trocar placement has been proposed to improve access of vitreoretinal instruments to the retinal surface. We report two myopic eyes in which scleral transillumination was used intraoperatively to assess the posterior edge of the ciliary body and to guide trocar placement when needed. Case 1 underwent surgery for a macular hole, and Case 2 underwent surgery for macular hole retinal detachment. In both cases, involving the right eye, an intraocular light pipe was inserted through the superonasal trocar and directed toward the superotemporal sclera under reduced room and microscope illumination. The resulting transillumination boundary was marked externally. In Case 1, the trocar was placed at the conventional insertion site, and scleral transillumination was performed intraoperatively to assess the relationship between the transillumination boundary and the ciliary body. The boundary was located approximately 6 mm posterior to the corneal limbus, and intraoperative indentation confirmed that it corresponded to the posterior edge of the ciliary body. Postoperative anterior segment optical coherence tomography (AS-OCT) showed a ciliary body length of around 6.6 mm. In Case 2, scleral transillumination was used to guide trocar placement. The initial illumination border was observed approximately 6 mm posterior to the limbus and was used as a reference for trocar insertion. However, further peripheral observation revealed an additional posterior dark band approximately 9 mm from the limbus. External marking and indentation of this band corresponded to the posterior edge of the ciliary body, and postoperative AS-OCT showed a ciliary body length of around 9.9 mm. These preliminary observations from two highly myopic eyes suggest that scleral transillumination may be a useful intraoperative method for estimating the posterior edge of the ciliary body and guiding trocar placement. In Case 2, the initial transillumination boundary was located anterior to an additional peripheral dark band, which appeared to correspond more closely to the actual posterior edge of the ciliary body. Nevertheless, for practical vitrectomy, the anterior illumination boundary provided a sufficiently posterior and safe landmark for trocar placement. This technique should be considered selectively in highly myopic eyes with axial elongation rather than in eyes with normal or short axial length. Further studies are needed to determine the generalizability of these findings.

## Introduction

High myopia is a well-known risk factor for various vitreoretinal disorders, including macular hole, myopic traction maculopathy, rhegmatogenous retinal detachment (RRD), and macular hole retinal detachment (MHRD) [1-4]. Because of axial elongation and structural fragility of ocular tissues, surgical management in these eyes is often more technically challenging than in eyes with normal axial length [2,5]. In particular, during pars plana vitrectomy (PPV), the elongated globe alters the spatial relationship between the trocar insertion site and the posterior pole, which may make surgical manipulation at the macula more difficult [5].

In standard PPV, trocars are typically inserted 3-4 mm posterior to the corneal limbus to avoid creating iatrogenic retinal breaks [6]. However, in highly myopic eyes, this conventional distance may position the surgical ports relatively anterior to the optimal location. Consequently, instruments such as membrane forceps may not sufficiently reach the macular region, potentially compromising delicate maneuvers at the posterior pole [5,7]. This issue may be particularly relevant when delicate macular procedures, such as internal limiting membrane (ILM) peeling, require stable instrument positioning at the posterior pole. To address this issue, modified trocar placement using a more posterior entry site has been reported based on the observation that the pars plana in highly myopic eyes is longer than that in normally proportioned eyes [8,9]. Other approaches to facilitate vitrectomy in highly myopic eyes include confirming the pars plana using intraoperative endoscopic imaging or employing modified surgical instruments such as long-shaft designs [10,11]. However, these methods may require specialized equipment or non-routine instruments, which may limit their practical use in some surgical settings.

In this report, we describe a scleral transillumination technique to intraoperatively estimate the pars plana and guide trocar insertion in myopic eyes. By enabling safer posterior positioning of the trocar, this technique may facilitate membrane peeling at the posterior pole even with standard-shaft instruments, without requiring additional equipment.

## Case presentation

Case 1

**Figure 1:**
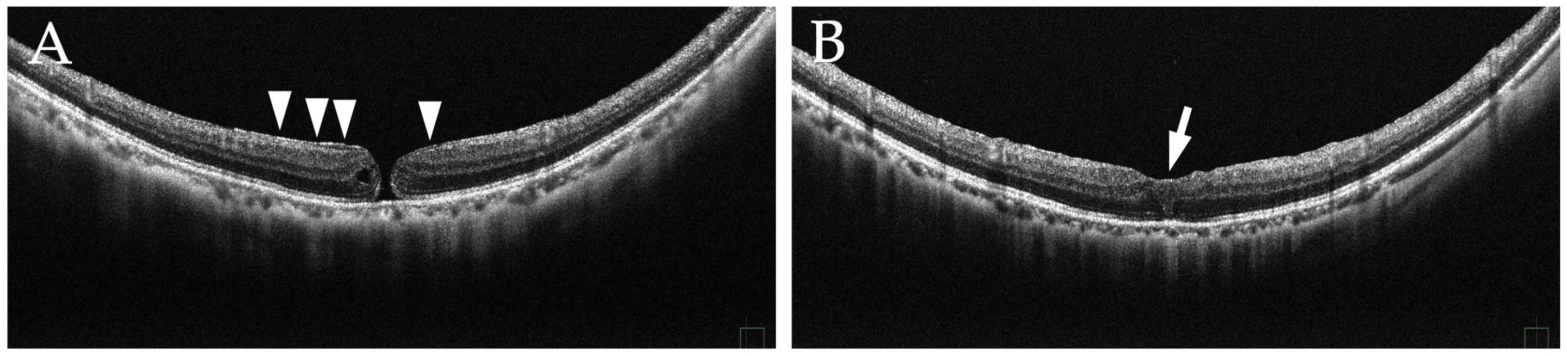
Preoperative and postoperative vertical OCT in Case 1. (A) Preoperative vertical OCT shows a full-thickness macular hole with epiretinal proliferation (arrowhead). (B) Two-week postoperative vertical OCT shows closure of the macular hole (arrow). OCT: optical coherence tomography.

A 66-year-old man presented to a local clinic complaining of decreased vision in the right eye (RE) that had persisted for one month. He was referred to our institution after being diagnosed with macular hole RE. He had previously undergone PPV for RRD in both eyes (BE); eight years earlier, RE and one year in the left eye (LE) before the current presentation. At the initial visit, best-corrected visual acuity (BCVA) was 0.8 RE and 1.2 LE. Intraocular pressure (IOP) was 20 mmHg BE. Axial length was 27.71 mm RE and 27.57 mm LE. Fundus examination revealed a macular hole with epiretinal proliferation RE (Figure 1A).

**Figure 2:**
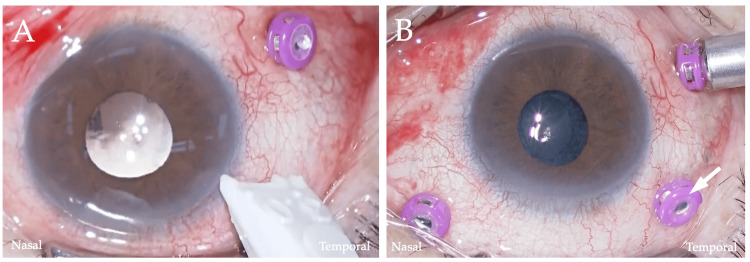
Intraoperative anterior segment views in Case 1. (A) A scleral marker is used to identify the superotemporal trocar insertion site, approximately 3-4 mm posterior to the corneal limbus. (B) A superotemporal trocar is inserted approximately 3.5 mm posterior to the corneal limbus (arrow).

A 25-gauge three-port PPV was performed using the Constellation Vision System (Alcon, Fort Worth, TX, USA), under sub-Tenon anesthesia using 2% lidocaine. The initial scleral trocars were placed approximately 3.5 mm posterior to the corneal limbus (Figure 2A, 2B). ILM peeling and epiretinal proliferation embedding were performed. Fluid-air exchange was then performed, and 0.6 cc of 100% sulfur hexafluoride gas was injected at the end of surgery. At two weeks postoperatively, optical coherence tomography (OCT, RS-3000 Advance, NIDEK, Gamagori, Japan) confirmed closure of the macular hole (Figure 1B).

Before fluid-air exchange, scleral transillumination was performed using a light pipe inserted through the superonasal port and directed toward the superotemporal sclera (Video 1).

**Video 1:** Scleral transillumination observation in Case 1.

**Figure 3:**
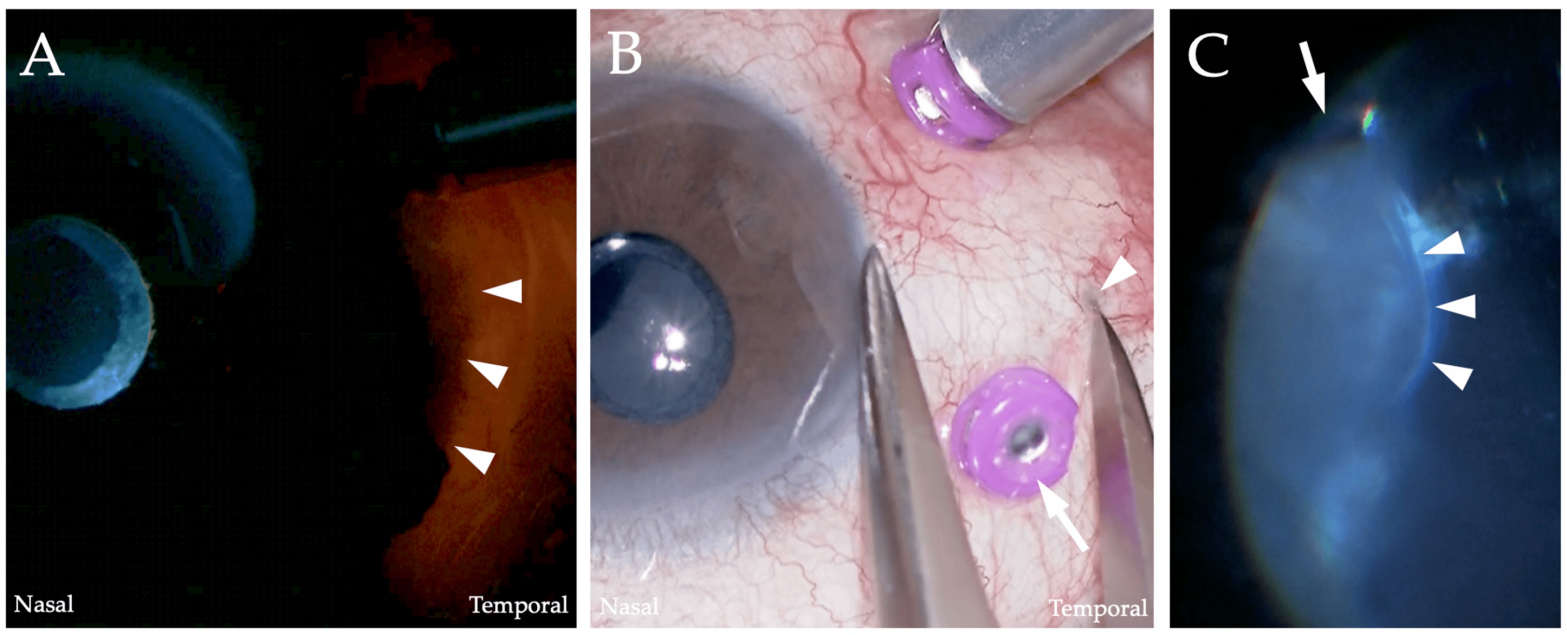
Estimation of the posterior edge of the ciliary body using scleral transillumination observation in Case 1. (A) Intraocular illumination with a light pipe directed from the superonasal port toward the superotemporal sclera reveals a difference in scleral transillumination intensity (arrowhead). (B) The border between the transilluminated area and the non-transilluminated area is marked externally (arrowhead). The straight-line distance from the corneal limbus to the marked site is approximately 6 mm. The marked site lies posterior to the superotemporal trocar site (arrow). (C) The marked site is confirmed under scleral indentation using a wide-angle viewing system and corresponds to the posterior edge of the ciliary body (arrowhead). The photograph is shown as a non-inverted view.

During the procedure, room illumination was reduced, and the operating microscope (Lumera 700, Carl Zeiss Meditec AG, Jena, Germany) light was turned off. Under these conditions, a clear boundary of scleral translucency was observed posterior to the existing trocar site (Figure 3A). The straight-line distance from the corneal limbus to this boundary, measured using a surgical caliper, was approximately 6 mm (Figure 3B). The boundary was grasped with fine forceps (M-5R, Inami, Tokyo, Japan) and gently indented, and observation under a wide-angle viewing system (RESIGHT, Carl Zeiss Meditec AG) confirmed that the indentation corresponded to the posterior edge of the ciliary body (Figure 3C). Postoperatively, anterior segment assessment of the ciliary body was performed using anterior segment-OCT (AS-OCT, CASIA2, Tomey Corp., Nagoya, Japan). The posterior edge was defined as the point where the outer contour extending from the scleral spur (SS) and the inner contour of the ciliary body converged, with no visible gap between the ciliary body and the sclera [12,13].

**Figure 4:**
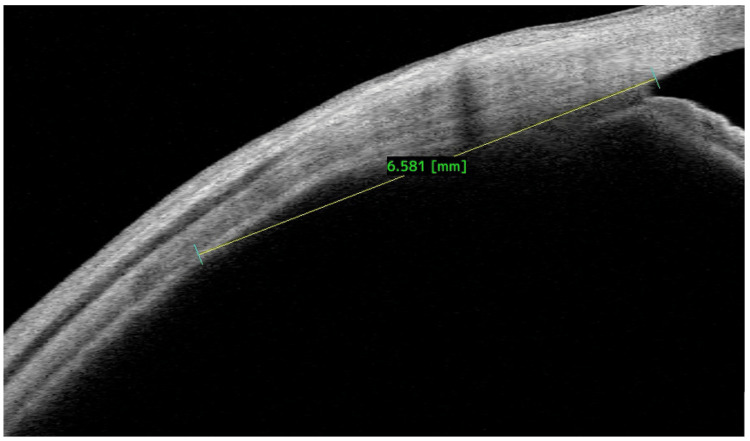
Anterior segment OCT in Case 1. The straight-line distance from the SS to the posterior edge of the ciliary body is 6.581 mm. The posterior edge of the ciliary body is defined as the peripheral convergence point of the outer line traced from the SS and the inner line traced from the ciliary body. OCT: optical coherence tomography; SS: scleral spur.

Using the built-in measurement software, the ciliary body length was 6.581 mm in this case, which was generally consistent with the intraoperative straight-line measurement from the corneal limbus to the transillumination boundary (Figure 4A). In this case, the surgery was completed uneventfully using the standard procedure, and scleral transillumination could be successfully confirmed intraoperatively.

Case 2

**Figure 5:**
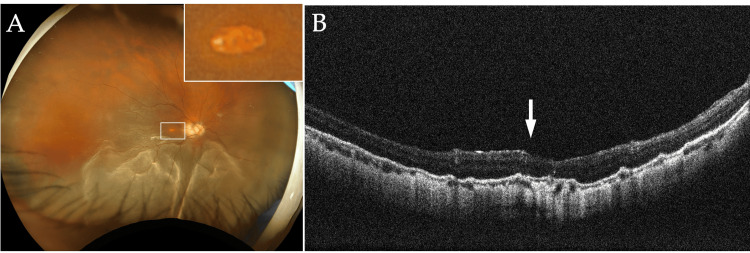
Widefield fundus photographs and postoperative vertical OCT in Case 2. (A) Preoperative widefield fundus photograph shows a macular hole retinal detachment. The macular hole is highlighted in the magnified inset. (B) Three-month postoperative vertical OCT shows retinal reattachment and closure of the macular hole (arrow). OCT: optical coherence tomography.

A 60-year-old man with a history of intellectual disability presented to a local clinic with complaints of decreased vision. Because of communication difficulties, the laterality and onset of symptoms were unclear. He was referred to our institution after being diagnosed with MHRD RE and cataracts BE. At presentation, BCVA was 0.05 RE and counting fingers LE. IOP was 9 mmHg RE and 12 mmHg LE. Axial length was 28.69 mm RE and 30.57 mm LE. Fundus examination revealed total retinal detachment associated with a macular hole RE (Figure 5A).

**Figure 6:**
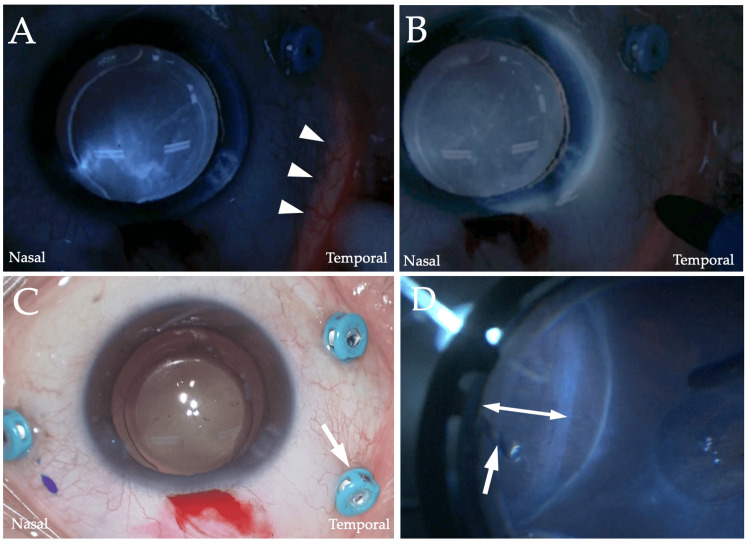
Intraoperative findings during the initial scleral transillumination observation in Case 2. (A) An anterior segment image obtained during the initial scleral transillumination observation shows the border of the transilluminated area (arrowhead). (B) The border is marked externally, and the straight-line distance from the corneal limbus to the marked site is approximately 6 mm. (C) A superotemporal trocar is inserted 0.5 mm anterior to the marked border as a safety margin (arrow). (D) Under a wide-angle viewing system, the posterior edge of the ciliary body (arrowhead) is identified more than 0.5 mm posterior to the superotemporal trocar insertion site, as indicated by the double-headed arrow. The photograph is shown as a non-inverted view.

Combined cataract surgery and 25-gauge three-port PPV using the Constellation Vision System (Alcon) was performed RE. Because of the patient’s intellectual disability, surgery was performed under general anesthesia. Before insertion of the superotemporal scleral trocar, initial scleral transillumination observation was performed (Figure 6A, Video 2). The procedure was performed as described in Case 1. The boundary of the transilluminated area was marked externally (Figure 6B), and the straight-line distance from the corneal limbus to the mark was approximately 6 mm. A superotemporal trocar was inserted 0.5 mm anterior to the mark (Figure 6C). An ILM inverted flap was created, subretinal fluid was drained through an intentional retinal break, fluid-air exchange was performed, and surgery was completed with silicone oil tamponade. No complications related to scleral transillumination were observed. Three months after the initial surgery, silicone oil removal was performed RE. The retina remained attached, and the macular hole was closed (Figure 5B).

**Video 2:** Initial and repeated scleral transillumination observation in Case 2.

**Figure 7:**
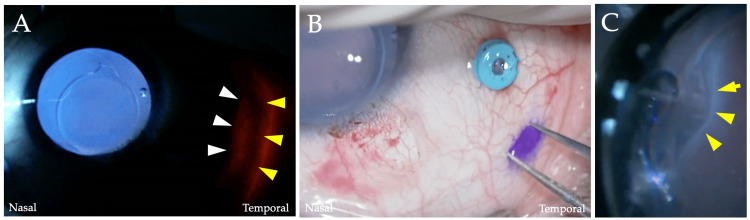
Intraoperative findings during repeated scleral transillumination observation in Case 2. (A) Repeated scleral transillumination observation extending to a more peripheral area reveals another dark band (yellow arrowhead) posterior to the initial border (white arrowhead). (B) The peripheral dark band is marked externally and grasped with forceps. The straight-line distance from the corneal limbus to the marked site is approximately 9 mm. (C) Under a wide-angle viewing system, the grasped site corresponds to the posterior edge of the ciliary body (yellow arrowhead). The photograph is shown as a non-inverted view.

Intraoperatively, wide-angle viewing (RESIGHT, Carl Zeiss) of the superotemporal trocar insertion site showed that, although the ora serrata was expected to be located approximately 0.5 mm posterior to the insertion site, it was actually located several millimeters further posteriorly (Figure 6D). Therefore, repeated scleral transillumination observation was performed (Video 2). On peripheral observation, a dark band was identified peripheral to the initial boundary (Figure 7A). The anterior edge of this dark band was marked externally (Figure 7B). The straight-line distance from the corneal limbus to this mark was approximately 9 mm. When the mark was grasped with fine forceps (M-5R, Inami) and the fundus was observed, the grasped site corresponded to the posterior edge of the ciliary body (Figure 7C).

**Figure 8:**
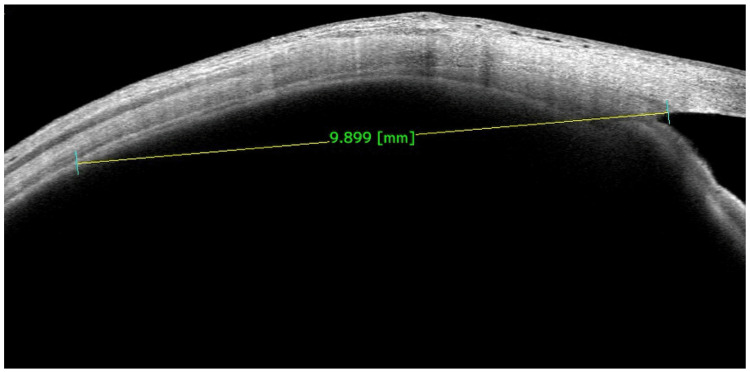
Three months postoperative anterior segment OCT in Case 2. The straight-line distance from the SS to the posterior edge of the ciliary body is 9.899 mm. The posterior edge of the ciliary body is defined as the peripheral convergence point of the outer line traced from the SS and the inner line traced from the ciliary body. OCT: optical coherence tomography; SS: scleral spur.

Postoperatively, anterior segment assessment of the ciliary body was performed using AS-OCT (CASIA2, Tomey Corp). As in Case 1, the straight-line distance from the SS to the posterior edge of the ciliary body was measured using the built-in measurement software. The ciliary body length was 9.899 mm in this case, which was generally consistent with the intraoperative straight-line measurement from the corneal limbus to the transillumination boundary (Figure 8).

## Discussion

Our report showed the following two findings. First, in myopic eyes, scleral transillumination may be used to estimate the posterior edge of the ciliary body. Second, in some cases, a dark band may be observed peripheral to the anterior illumination border, and this band may correspond more closely to the posterior edge of the ciliary body. Accurate localization of the posterior border of the ciliary body is clinically important during pars plana trocar insertion, particularly in highly myopic eyes, because excessively anterior placement may increase the risk of ciliary body injury, whereas excessively posterior placement may increase the risk of retinal injury. In highly myopic eyes, anatomical variation of the pars plana may further complicate estimation of a safe entry site [14].

First, scleral transillumination may help estimate the posterior edge of the ciliary body in myopic eyes. As noted above, axial elongation in myopic eyes can limit instrument access to the posterior pole, whereas elongation of the pars plana may allow more posterior trocar placement when appropriate [8]. Scleral transillumination has previously been used to identify the ciliary body for safe sclerotomy or intravitreal injection in infants and for localization of the ciliary body during cyclodiode laser treatment procedures in glaucoma patients [15,16]. These findings support the concept that differences in light transmission through the anterior uveal tissue can provide a practical intraoperative landmark for estimating the posterior extent of the ciliary body. Variations in scleral thickness, choroidal pigmentation, and ciliary body configuration may influence local light transmission properties and thereby contribute to the visibility of the transillumination border.

In the present report, both cases involved the right eye; therefore, the superotemporal scleral transillumination site was located on the surgeon’s dominant-hand side. The temporal site is generally more feasible than the nasal site for scleral transillumination, as the temporal conjunctiva and sclera can usually be exposed more widely. In the left eye, however, the corresponding temporal site is positioned on the surgeon’s non-dominant side for a right-handed surgeon. Although this may be acceptable for surgeons accustomed to working from the non-dominant side, nasal scleral transillumination may be considered when access from the dominant-hand side is preferred. Nevertheless, nasal transillumination may be less reliable in some cases. Thus, the use of this technique should be tailored according to eye laterality, the surgeon’s handedness, and the surgeon’s familiarity with non-dominant-side manipulation.

Second, in some cases, a dark band may be observed peripheral to the anterior illumination border, and this band may correspond to the posterior edge of the ciliary body. In Case 2, wide-angle fundus observation showed a white line at the posterior edge of the ciliary body (Figure 6D). This white line may correspond to the dark band observed during scleral transillumination. Anatomically, the white line represents cystoid changes in the peripheral retina near the ora serrata [17,18]. Peripheral cystoid degeneration consists of small intraretinal microcystoid cavities and is most frequently seen in the temporal peripheral retina [17,18]. Whether this band is visible may depend on differences in tissue translucency. In the two cases in this report, AS-OCT did not show an obvious difference in ciliary body thickness. The appearance of the band cannot be fully predicted from tissue thickness alone. Other factors, such as pigmentation, scleral or ciliary body tissue density, and the degree of peripheral retinal cystoid change, may also be involved. Even when this band is observed, trocar insertion at the anterior illumination border may be safe and may provide a sufficient distance from the corneal limbus. Nevertheless, recognition of this band may improve the surgeon’s anatomical understanding during trocar planning and may provide supplementary intraoperative information regarding the posterior extent of the pars plana in selected highly myopic eyes.

In our experience, scleral transillumination may be less reliable for evaluating the pars plana in eyes with normal axial length. This may be because myopia-related structural changes in the ocular wall, such as scleral and choroidal thinning and remodeling of scleral collagen architecture, are less pronounced than in highly myopic eyes. In pediatric eyes and eyes with short axial length, these anatomical differences may be even more marked, with a relatively thicker sclera and a shorter pars plana [12]. Alternative methods should therefore be considered for determining a safe trocar entry site in these eyes. In addition, in eyes with normal axial length, trocars are usually placed 3-4 mm posterior to the corneal limbus, and more posterior placement generally offers little benefit. Thus, scleral transillumination may have limited practical value in eyes with normal axial length. Therefore, the clinical usefulness of scleral transillumination may be greatest in eyes with marked axial elongation, in which conventional limbus-based estimation alone may be less reliable.

The present observations regarding transillumination-assisted localization of the posterior border of the ciliary body were based on only two myopic eyes. Therefore, the generalizability of these findings remains limited. Further clinical studies involving larger numbers of eyes and analyses incorporating axial length and other ocular biometric parameters will be necessary to clarify the applicability and reproducibility of this technique across different anatomical conditions.

## Conclusions

Scleral transillumination may be a useful intraoperative method for estimating the posterior edge of the ciliary body in myopic eyes. In some cases, an additional peripheral dark band may be observed and may correspond more closely to the posterior edge of the ciliary body. For practical vitrectomy, the anterior illumination border may provide a sufficiently posterior and safe landmark for trocar placement. This technique should be considered selectively for highly myopic eyes, not for eyes with normal or short axial length. As these findings are based on only two cases, further studies are required to confirm their generalizability and clinical applicability.
